# Why are we fat? Discussions on the socioeconomic dimensions and responses to obesity

**DOI:** 10.1186/1744-8603-6-7

**Published:** 2010-04-23

**Authors:** Geof Rayner, Mabel Gracia, Elizabeth Young, Jose R Mauleon, Emilio Luque, Marta G Rivera-Ferre

**Affiliations:** 1 City University, London, UK; 2 Universidad Rovira i Virgili, Tarragona, Spain; 3 University of Staffordshire, Staffordshire, UK; 4 Universidad del País Vasco UPV/EHU, Vitoria, Spain; 5 Universidad Nacional de Educación a Distancia, Madrid, Spain; 6 Universidad Autónoma de Barcelona, Bellaterra, Spain

## Abstract

This paper draws together contributions to a scientific table discussion on obesity at the European Science Open Forum 2008 which took place in Barcelona, Spain. Socioeconomic dimensions of global obesity, including those factors promoting it, those surrounding the social perceptions of obesity and those related to integral public health solutions, are discussed. It argues that although scientific accounts of obesity point to large-scale changes in dietary and physical environments, media representations of obesity, which context public policy, pre-eminently follow individualistic models of explanation. While the debate at the forum brought together a diversity of views, all the contributors agreed that this was a global issue requiring an equally global response. Furthermore, an integrated ecological model of obesity proposes that to be effective, policy will need to address not only human health but also planetary health, and that therefore, public health and environmental policies coincide.

## Introduction

Why are we getting fat? Is it the result of our new-found freedom to consume cheap foods and be less physically active or are there less obvious factors which help explain the world-wide rise in obesity? While much remains uncertain about the causes of population weight gain, what we do know is that, beginning in the USA then spreading to Europe, obesity is fast emerging as the new pandemic of the XXI^st ^century [1-3], that social and health costs associated with obesity continue to rise [4] and that in some developing countries obesity is rising fast [5]. The problem of obesity is both real and seems to be getting worse. In the USA it has been suggested that more than 50 per cent of the adult population will be obese by 2030 [6].

Obesity presents a particular challenge for public health policy because treatment is expensive, with poor results and a marginal impact on population trends, suggesting that the emphasis must be placed on prevention [7,8]; but prevention efforts have been shown so far to have been relatively ineffective [9]. Obesity can also be characterised as a public policy problem since public policy may itself has a specific role in promoting - or at least failing to restrict - the determinant factors underlying population weight change [10]. Socioeconomic dimensions of global obesity, including those factors promoting it, those surrounding the social perceptions of obesity and those related to public health solutions, were discussed at the European Science Open Forum (ESOF) 2008 in Barcelona, Spain. ESOF is an independent arena for open dialogue and exchange of ideas on the role of sciences in society, offering a platform for cross-disciplinary interaction and communication on current and future trends http://www.euroscience.org. This short paper by the contributors to ESOF discussions presents their summary views on the socioeconomic dimensions of this important public health topic, including new holistic approaches to tackle this health issue.

Obesity occurs when a person's Body Mass Index (BMI), calculated as the weight (kgs) divided by the square height (cm), exceeds 30. For children, issues of measurement are more complex; nor is BMI an always reliable measure given diversity of body shape. Obesity is also a cultural matter [11]. While some societies find large body size acceptable, even an aspirational goal, this is not commonly so in Western Societies [12]. In Europe, where obesity is likely to have negative connotations in any language, it is increasing in both absolute and relative terms and has been shown to be linked to a variety of social determinants [13].

Population weight gain is increasing despite the best efforts of the health authorities to inculcate healthy eating habits or the ubiquity of commercial weight-loss and low-fat food products. This suggests that an analysis of obesity requires more than an understanding of individual dietary patterns but needs to engage with a more complex explanation incorporating the recognition of the paradox that while society may discourage fatness discursively, it might also encourage it in practice.

Obesity has been classified by the World Health Organization (WHO) as a non-communicable disease (although it might be better described as an 'avoidable chronic illness') [14]. WHO's expert guidance on obesity causation is found in the joint WHO/Food and Agriculture Organisation report TRS 916 [15] and this analysis was ratified at the 2004 World Health Assembly [16]. The WHO approach provides a powerful understanding of causation, especially when allied with general explanations of the historical development of obesogenic drivers known as the Nutrition Transition [17-20]. Unfortunately, scientific explanations of obesity carry less weight in the media than behavioural and biomedical discourses that emphasise immediate (or 'proximate') causation and individual responsibility, reflecting what some have seen as the reductionist tendency in the prevalent 'western model' of health [21].

## Discussion

### Variables affecting Obesity: The Spanish case

Weight gain does not affect everybody in the same way. Not everyone who is overweight is ill because of it and not everyone who is overweight has a poor diet. The way in which people consume food and manage their health varies according to many factors ranging from socioeconomic status, gender, age and ethnic origin as well as the interaction between micro- and macro-structural factors that change from one society to another [22].

Across the European continent, obesity has been growing in prevalence, with particular concern focused on children [13]. The annual rate of increase appears to be upward; from around 0.2% during the 1970s to 0.8% in the early 1990s [3]. There are important differences within and between countries. Croatia and Finland have the highest prevalence among males older than 15 years (around 22%), while Uzbekistan and Norway have the lowest prevalence (around 6%). The relative importance of the specific factors which explain such wide variance are difficult to establish since national wealth, local dietary patterns, culture and other factors which appear to be driving this trend, appear to interact in complex ways.

**Figure 1:**
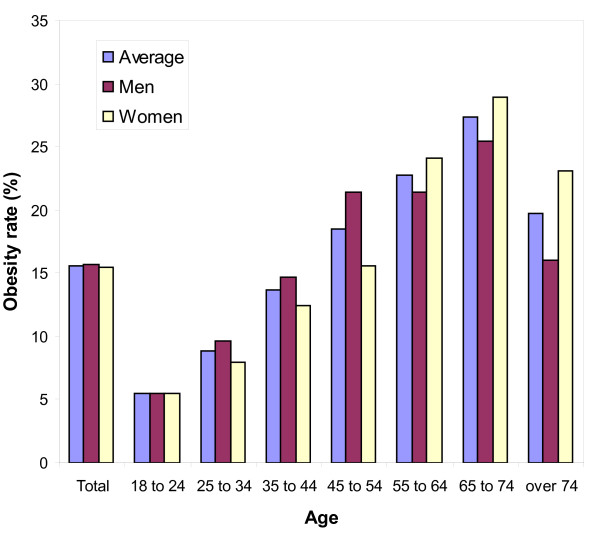
**Obesity rates among adults in Spain in 2006, by sex**.

In Spain, the location of the ESOF meeting, variables such as age, social class, sex, and region of habitation, for example, all appear to be related to obesity prevalence [23]. Overall prevalence of obesity among adults is 15.5% (15.7 and 15.4% among men and women, respectively; Figure 1), and this percentage increases with the age, from 5.5% at 18-24 years old to 27.3% at 65-74 years. With respect to social class it increases from 10.4% among the highly skilled to 19.5% among the unskilled. In the case of women, it increases threefold from 6.9% among the highly skilled to 21.8% for unskilled. Pensioners have the highest rate of obesity (23%) followed by homeworkers (20.6%). Differences also expressed regionally. The prevalence ranges from 11%-12% in La Rioja, Madrid and the Balearic Islands to 18%-19% in Murcia, Andalusia or Extremadura [23].

**Figure 2:**
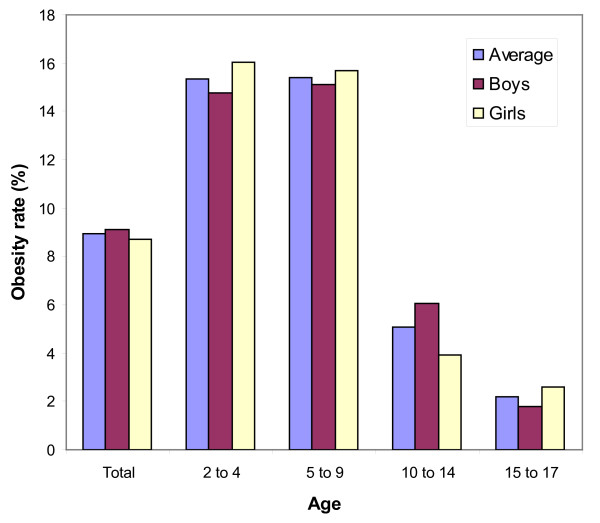
**Obesity rates among Spanish children in 2006, by sex**.

Among the Spanish children (2 to 17 years old) the numbers are also of growing concern (Figure 2). The highest prevalence is for children between 5 to 9 years old (15.4%) and those in the range of 2 to 4 years old (15.3%). Parents' professional background is related to prevalence, increasing from 4.4% for the more skilled to 12.2 and 11.4% for those less skilled. In terms of territorial differences, the highest prevalence is in the Canary Islands, Ceuta and Melilla, Comunidad Valenciana, La Rioja and Andalusia (from 12 to 16%) and the lowest rates are found in Asturias, Castilla la Mancha, Galicia, Madrid and Basque Country (from 4.5 to 5.5%) [23].

### Health system responses to obesity

It is a metabolic rule that when we consistently overeat we put on weight: it therefore follows that dietary intake and physical activity matters [24]. However, changes in diet and levels of physical activity always occur in historical and social context, a fact which is often ignored [25]. The result is that behavioural and biomedical discourses emphasise proximate causation and individual responsibility [26], which, in turn, limits the possibilities of public health action [27]. Proximate causes include dietary patterns, levels of physical activity, and genetic factors. Specialists stress the consequences of reductions that have occurred in everyday activity, such as walking, the time spent in sedentary pursuits and patterns of body weight in parent and child. Advice from experts therefore centres on changing 'disordered' lifestyles and consumption patterns in order to promote healthy habits, guided by general practitioners, pharmacists and others, increasing the responsibility of individuals through taming their appetites and encouraging the self-regulation of appetite. It follows that explanations of obesity found in the media, and refracted into political discourse, focus on a person's choices and lifestyle in their immediate environment (behavioural frame), although lately there is an increasing recognition of the importance of the environmental factors which simultaneously constrain mobility and stimulate the intake of high fat, high energy foods (systemic frame) [27]. As a consequence, policies focus on the individual and highlight the role of individual choice in diet or participation in physical activity as the key to health improvement. The principal recommended mechanism of change is educational, including family recognition that the problem actually exists, and encouragement towards healthy lifestyles, along with some environmental improvements, such as food labelling [28].

Given that there is little evidence that the favoured social marketing approach, in any of its different formats, has halted overall obesity trends [29,30], the question might be as to why the focus on educating individuals remains dominant to prevention efforts. The answer may be that alternative explanations, which by implication require a much broader range of policies and actions, are too economically and politically challenging.

### Markets, technology and medicalization of obesity

In more market-oriented societies, it has been suggested that social and health problems take on a more common character [31]. In the case of obesity there are numerous commercial opportunities. For instance, functional foods, dietary advice and diet book publishing have risen rapidly. In the UK, the sale of functional foods has risen from £134 million in 1998 to £1.7 billion in 2007 [32]. While large investments have been made by pharmaceutical companies in the field of anti-obesity drugs, market growth does not follow the growth of obesity prevalence but rather the scope of reimbursement for pharmacological management, which in many countries remains limited [33].

To the dietary recommendations launched by health professionals and public authorities to promote health, the numerous and often contradictory messages distributed through market channels must be added [34]. Advice in favour of the optimum diet and normal body weight has been adopted by the health and 'body care' market. Advertising and marketing campaigns offer clues for understanding the role of the food industry, aided by scientific and technological innovations. The marketing industry is the creator, par excellence, of the rhetoric of "well-being" and the commercialization of the term "health", an umbrella concept that subsumes a broad range of other concepts: pleasure, beauty, convenience and mental health [35]. Products are advertised as "light" or "free" - as in cholesterol-free, sugar-free, and fat-free. In the same way, products "with" - fibre, lactic acid bacteria, minerals, fatty acids -represent a new generation of products designed to cater to our perception of well-being and health [36].

For all these reasons, it is difficult to separate interests related to health and interests related to commerce in biomedical discourse [37]. Thus, at the same time that the medical establishment warns against overweight as a threat to health, the consumer and medical economy is inundated with food products of doubtful nutritional quality, diet plans, weight loss drugs and weight loss surgery.

### Structural causes, structural solutions

Is consumer choice driving global or national trends in population weight gain, and underlying the independent consumption decisions of millions of people, or are there other, less visible, but nevertheless real, explanatory factors at work? While individual consumer choice provides the enduring narrative circulated in policy circles and the media, attention to structural causes (and thus structural solutions) linked to the economic and cultural identity of society is given less prominence. Structural types of explanation for obesity focus on long-term economic and social policy trends - some of which take a global form [31] and the changing context of consumer choices.

One approach for explanation begins by analysing changes within the food supply chain and the reshaping of the way in which food is produced, formulated, priced, marketed and consumed. Following the lead of the USA, farming legislation has resulted in ever-cheaper basic ingredients available to food manufacturers and retailers, boosting portion sizes as well as consumption of high fat, energy dense foods [38]. Since the 1970s the European Common Agricultural Policy, has balanced subsidies to primary producers with economic liberalisation in markets, particularly in food manufacturing, food services and food retailing. Whereas the former boosted production levels, the latter introduced new capital into the food manufacturing and retail segments, profoundly altering food provisioning systems [39]. The impact has been dramatic. Traditional diets, in particular 'Mediterranean Diet' of Spain, Italy, Greece and Malta - previously much praised by nutritionists - has given way to new dietary regimes containing much higher levels of saturated fats, salt and sugars. Such trends extend well beyond Europe and may be resulting in a global culture of food [40]. Even so, apparently homogenizing forces produce outcomes which vary according to national or cultural context [41].

A second type of policy analysis aims at encompassing these economic and business realities, public policy and cultural factors, implying the need for scientific collaboration across different research disciplines (as well as co-ordination and collection of different types of knowledge) together with detailed understanding of the interplay of local, regional, national and global factors [42-44]. A third focus points to the imbalance of power between the public good and corporate freedom [45] and raises questions over current understandings of health and the nature of the economical interventions needed to support health [46].

If these perspectives add to our knowledge of structural factors, there remains open the need to construct an integrated and holistic perspective which can draw upon not only the biological and physiological aspects of obesity and its social, economic and environmental determinants, but which also examines the feedback between the conditions which are shown to influence health to those which affect the natural environment. In part, the formulation of this new approach has already begun. An ecological perspective has already been established within the prestigious US Institute of Medicine [47], while a specifically ecological model was formulated to examine obesity [48]. An ecological approach is also present within the British government's Foresight Study of obesity [49]. It has been suggested that tackling obesity and tackling climate change can both be characterised as 'ecological' in form and share a number of similar underlying drivers and characteristics [29,50]. Both have been years in the making, both involve the interplay between similar factors - overuse of energy derived from fossil fuels and underutilisation of human energy, overproduction and waste, and lack of sustainability- and both, in public policy terms, are insufficiently recognised and require long term framework of action, implying a thorough redirection of society. It is agreed that steps towards low-carbon living (including changes in consumption patterns or green-designed cities) have health benefits that will improve quality of life by challenging diseases arising from affluent high-carbon societies, such as obesity [50]. A full response requires a holistic global approach, but this fact should not be a reason to delay changes that are beneficial to human health and can be implemented immediately [50].

## Conclusions

Obesity has been dramatised as one of the leading public health challenges of our age, but it is equally a conceptual and public policy challenge as well. What emerged from the ESOF scientific table is that solutions presented at the level of the individual, whether they be health education or medical interventions, are unlikely to be successful while newer ecological approaches have yet to capture the attention of policy makers. As the societal consequences of obesity fully emerge, pressure will be placed upon supply chains, economic actors and upon public and private behaviour to make wholesale changes.

## Competing interests

The authors declare that they have no competing interests.

## Authors' contributions

MG, LY, JRM and EL have written the summarised ideas of their contribution to the ESOF conference. GR has written the summarised ideas of his contribution to the ESOF conference and helped in the writing of the paper bringing all the ideas together. MGRF organised the conference and led the writing of the paper. All authors have read and approved the final manuscript.

## References

[B1] Wang Y Beydoun MA The Obesity Epidemic in the United States-Gender, Age, Socioeconomic, Racial/Ethnic, and Geographic Characteristics: A Systematic Review and Meta-Regression Analysis Epidemiological Reviews 2007 29 6 28 10.1093/epirev/mxm007 17510091

[B2] WHO Obesity: preventing and managing the global epidemic. Report of a WHO Consultation Obesity: preventing and managing the global epidemic. Report of a WHO Consultation 2000 Geneva: World Health Organisation 11234459

[B3] WHO The challenge of obesity in the WHO European Region 2005 Copenhagen: World Health Organisation

[B4] Rayner G Rayner M Fat is an economic issue: combating chronic diseases in Europe Eurohealth 2003 9 17 20

[B5] Pan H Jiang Y Jing X Fu S Jiang Y Lin Z Sheng Z Cole TJ Child body mass index in four cities of East China compared to Western references Annals of Human Biology 2009 36 98 109 10.1080/03014460802575641 19085513 PMC2645134

[B6] Wang Y Beydoun MA Liang L Caballero B Kumanyika SK Will All Americans Become Overweight or Obese? Estimating the Progression and Cost of the US Obesity Epidemic Obesity 2008 16 2323 2330 10.1038/oby.2008.351 18719634

[B7] Sturm R Ringel J Andreyava R Increasing Obesity Rates and Disability Trends Health Affairs 2004 23 1 7 10.1377/hlthaff.23.2.199 15046144

[B8] Thompson D Edelsberg J Colditz GA Bird AP Oster G Lifetime Health and Economic Consequences of Obesity Arch Intern Med 1999 159 2177 2183 10.1001/archinte.159.18.2177 10527295

[B9] Summerbell CD Waters E Edmunds LD Kelly S Brown T Campbell KJ Interventions for preventing obesity in children Cochrane Database Systemic Reviews 2005 **Jul 20 (3)** 10.1002/14651858.CD001871.pub2 16034868

[B10] Lang T Rayner G Obesity: a growing issue for European policy? Journal of European Social Policy 2005 15 301 327 10.1177/0958928705057263

[B11] Gilman SL Fat: A Cultural History of Obesity 2008 Cambridge: Polity Press

[B12] de Garine I Pollock NJ (Eds) Social aspects of obesity 1995 London: Routledge

[B13] Branca F Nikogosian H Lobstein T (Eds) The challenge of obesity in the WHO European Region and the strategies for response 2007 Copenhagen: World Health Organisation

[B14] WHO Diet, Nutrition and the Prevention of Chronic Diseases Diet, Nutrition and the Prevention of Chronic Diseases 1990 Geneva: World Health Organisation

[B15] WHO/FAO Diet, Nutrition and the Prevention of Chronic Diseases: Report of a Joint WHO/FAO Expert Consultation Diet, Nutrition and the Prevention of Chronic Diseases: Report of a Joint WHO/FAO Expert Consultation 2003 Geneva: World Health Organisation/Food and Agriculture Organisation of the United Nations

[B16] WHO Fifty-seventh World Health Assembly 17-22 May WHA57.17 Global strategy on diet, physical activity and health 2004 Geneva: WHO

[B17] Popkin B The Nutrition Transition in Low-Income Countries: An Emerging Crisis Nut Rev 1994 52 285 298 10.1111/j.1753-4887.1994.tb01460.x 7984344

[B18] Drewnoski A Popkin B The Nutrition Transition: New Trends in the Global Diet Nut Rev 1997 55 31 43 10.1111/j.1753-4887.1997.tb01593.x 9155216

[B19] Popkin B An overview on the nutrition transition and its health implication: the Bellagio Meeting Public Health Nutrition 2001 5 93 103 10.1079/phn2001280 12027297

[B20] Popkin B The nutrition transation in the developing world Dev Policy Rev 2003 21 581 597 10.1111/j.1467-8659.2003.00225.x

[B21] MacDonald JJ Environments for Health: A salutogenic approach 2005 London: Earthscan

[B22] Debomy D The Europeans and Sustainable Food - Qualitative study in 15 European countries - Pan European Report 2006 Brussels: King Baudouin Foundation

[B23] Ministerio de Sanidad y Consumo National Health Survey 2006 Madrid 2006

[B24] Scheen AJ Luyckx FH Metabolic syndrome: definitions and epidemiological data Rev Med Liege 2003 58 479 484 14579611

[B25] Trowell H Burkitt D (Eds) Western Diseases: their emergence and prevention 1981 London: Edward Arnold

[B26] Inhorn MC Whittle KL Feminism meets the "new" epidemiologies: toward an appraisal of antifeminist biases in epidemiological research on women's health Social Science & Medicine 2001 53 553 567 10.1016/s0277-9536(00)00360-9 11478536

[B27] Lawrence RG Framing Obesity: The Evolution of News Discourse on a Public Health Issue The Harvard International Journal of Press/Politics 2004 9 56 75 10.1177/1081180X04266581

[B28] Andreasen A Marketing Social Change: Changing Behaviour to Promote Health, Social Development, and the Environment 1995 San Francisco CA: Jossey-Bass

[B29] Lang T Rayner G Overcoming Policy Cacophany on Obesity: an Ecological Public Health Framework for Politicians Obesity Reviews 2007 8 165 181 10.1111/j.1467-789X.2007.00338.x 17316322

[B30] Wymer W Rethinking the boundaries of social marketing: Activism or advertising? Journal of Business Research 2009 in press

[B31] Labonté R Schrecker T Globalization and social determinants of health: Introduction and methodological background Globalization and Health 2007 3 10.1186/1744-8603-3-5 PMC1924848 17578568

[B32] StrategyUnit Food: An Analysis of the Issues Food: An Analysis of the Issues. London 2008

[B33] Datamonitor Commercial and Pipeline Perspectives: Obesity - Lack of Reimbursement Limits Market Potential 2008 London: Datamonitor

[B34] Gracia M Comellas JM No comerás. Narrativas sobre comida, cuerpo y género en el nuevo milenio 2007 Barcelona: Icaria

[B35] Díaz Rojo JA Moranti i Marco R Westall Pixton D El culto a la salud y la belleza: la retórica del bienestar 2006 Madrid: Biblioteca Nueva

[B36] Hubert A Introduction Corps de femmes sous influences Questionner les normes 2004 10 l'Ocha Cd 5 11 (141pp)

[B37] Foucault M Historia de la sexualidad 1989 México: Siglo XXI Editores

[B38] Institute for Agriculture and Trade Policy The Farm Bill and Public Health: An Overview 2007 Minneapolis, MN: Institute for Agriculture and Trade Policy

[B39] Rayner G Barling D Lang T Sustainable Food Systems in Europe: policies, realities and futures Journal of Hunger and Environmental Nutrition 2008 3 145 168 10.1080/19320240802243209

[B40] Rayner G Hawkes C Lang T Bello W Trade liberalisation and the diet transition: a public health response Health Promotion International 2006 21 67 74 10.1093/heapro/dal053 17307959

[B41] Hawkes C Uneven dietary development: linking the policies and processes of globalization with the nutrition transition, obesity and diet-related chronic diseases Globalization and Health 2006 2 18 10.1186/1744-8603-2-4 PMC1440852 16569239

[B42] Rivera-Ferre MG The future of agriculture: Agricultural knowledge for economically, socially and environmentally sustainable development EMBO reports 2008 9 1061 1066 10.1038/embor.2008.196 18927582 PMC2581849

[B43] Bryant T Role of knowledge in public health and health promotion policy change Health Promotion International 2002 17 89 98 10.1093/heapro/17.1.89 11847142

[B44] Douglas F Greener J Teijlingen Ev 'Ask Me Why I'm Fat!' The Need to Engage with Potential Recipients of Health Promotion Policy to Prevent Obesity The Australian Economic Review 2008 41 72 77 10.1111/j.1467-8462.2008.00490.x

[B45] Young EM Globalisation and Food Security: novel questions in a novel context? Progress in Development Studies 2004 4 1 21 10.1191/1464993404ps073oa

[B46] Young EM World Hunger 1997 London: Routledge

[B47] Committee on Assuring the Health of the Public in the 21st Century Board on Health Promotion and Disease Prevention Institute of Medicine of the National Academies The Future of the Public's Health in the 21st Century 2002 Washington DC: The National Academies Press

[B48] Egger G Swinburn B An "ecological" approach to the obesity pandemic BMJ 1997 315 477 480 9284671 10.1136/bmj.315.7106.477 PMC2127317

[B49] Foresight Tackling Obesities: Future Choices 2007 London: Government Office of Science

[B50] Costello A Abbas M Allen A Ball S Bell S Bellamy R Friel S Groce N Johnson A Kett M Managing the health effects of climate change The Lancet 2009 373 1693 1733 10.1016/S0140-6736(09)60935-1 19447250

